# Fulminant Meningococcemia Presenting as Acute Abdomen Without Meningeal Signs: A Report of Two Cases and Review of Atypical Presentations

**DOI:** 10.7759/cureus.114264

**Published:** 2026-08-10

**Authors:** Anju Nabi, Zainab A Obaida, Rashid Nadeem, Rubina Monga, Rubina Lone, Hyfa Khan, Mohammed A Al Hakim, Maya Habous, Bhavna Mathur

**Affiliations:** 1 Laboratory Medicine/Microbiology, Dubai Health Authority, Dubai Hospital, Dubai, ARE; 2 Intensive Care Medicine, Dubai Hospital, Dubai, ARE; 3 Pathology and Laboratory Medicine/Microbiology, Rashid Hospital, Mohammed Bin Rashid University of Medicine and Health Sciences, Dubai, ARE; 4 Pathology/Microbiology, Al Jalila Children's Specialty Hospital, Mohammed Bin Rashid University of Medicine and Health Sciences, Dubai, ARE; 5 Medicine, Mohammed Bin Rashid University of Medicine and Health Sciences, Dubai, ARE; 6 Medicine, Independent Research, Dubai, ARE; 7 Pathology and Laboratory Medicine/Microbiology, Latifa Hospital, Mohammed Bin Rashid University of Medicine and Health Sciences, Dubai, ARE

**Keywords:** acute abdomen, atypical presentation, fulminant sepsis, meningococcemia, neisseria meningitidis, septic shock

## Abstract

Fulminant meningococcemia classically presents with meningitis or septicemia accompanied by rash and meningeal signs. However, atypical presentations dominated by gastrointestinal symptoms may delay diagnosis and increase mortality. We report two cases of microbiologically confirmed meningococcemia in young, previously healthy adults presenting primarily with acute gastrointestinal symptoms and abdominal pain, without rash or meningeal signs. The first case involved a 31-year-old man presenting with acute abdomen, septic shock, and multiorgan dysfunction who survived following early microbiological diagnosis and prompt antimicrobial therapy. The second case involved a 22-year-old man presenting with vomiting and septic shock who rapidly deteriorated and succumbed despite early resuscitation. Blood cultures in both cases grew *Neisseria meningitidis* serogroup W135.

## Introduction

Fulminant meningococcemia is a rapidly progressive and potentially fatal infection classically characterized by fever, headache, vomiting, and a petechial or purpuric rash. Early clinical recognition and prompt initiation of appropriate antimicrobial therapy are crucial to improving survival. However, it may present with uncommon and misleading manifestations, including abdominal pain, cough, arthritis, vasculitis, or pericarditis, which can obscure the diagnosis and delay life-saving treatment. Acute abdominal pain as the initial manifestation of meningococcal infection is rare and has been most frequently described in the right lower quadrant, often mimicking a surgical abdomen, particularly acute appendicitis [1]. Increasing reports describe atypical presentations lacking classical meningeal signs or rash, posing substantial diagnostic challenges for clinicians [2]. Meningococcal disease may also involve the peritoneum, presenting as mesenteric adenitis or peritonitis and further mimicking an acute surgical abdomen [3]. Abdominal pain as an atypical presentation of meningococcemia has also been described in adults [4], while other unusual manifestations, such as pericarditis, further illustrate its protean clinical spectrum [5]. Proposed mechanisms include mesenteric hypoperfusion, inflammatory enteritis, immune-mediated serositis, and microvascular thrombosis leading to peritoneal involvement [6]. Failure to recognize these atypical presentations may result in delayed antimicrobial therapy with catastrophic consequences. We describe two cases of fulminant meningococcemia in young adults who presented predominantly with acute abdominal symptoms in the absence of meningeal signs or rash, highlighting the importance of maintaining a high index of suspicion and the need for early microbiological evaluation in patients with unexplained acute abdomen and systemic illness.

## Case presentation

Case 1

A 31-year-old man presented in December 2025 with sudden-onset severe abdominal pain following suspected ingestion of contaminated food. Initial epigastric pain rapidly became generalized and radiated to the back in association with multiple episodes of vomiting and diarrhea. There was no recent travel, sick contact, or prior medical illness. At presentation, he was febrile, hypotensive, tachycardic, and clinically dehydrated. He was alert and oriented, with no neck stiffness, rash, focal neurological deficits, or photophobia. Abdominal examination revealed a tense abdomen with diffuse tenderness and guarding.

The laboratory investigation for this case is shown in Table 1.

**Table 1:** Laboratory investigations for Case 1 CRP: C-reactive protein

Parameter	Finding	Value / Description
White blood cell count	Leukocytosis	17.7 ×10⁹/L
Platelet count	Thrombocytopenia	129 ×10⁹/L
Coagulation profile	Coagulopathy	Abnormal
Renal function	Acute kidney injury	Creatinine 4.53 mg/dL
Lactate level	Lactic acidosis	9.26 mmol/L
CRP	Markedly elevated	259 mg/L
Procalcitonin	Markedly elevated	166 ng/mL

The microbiological investigation for this case is shown in Table 2.

**Table 2:** Microbiological investigation for Case 1

Flagged positive within 10 hours	Gram-negative diplococci observed on Gram stain
Positive	Neisseria meningitidis serotype W135 identified
Positive	Neisseria meningitidis detected
No growth	Not Applicable

**Figure 1:**
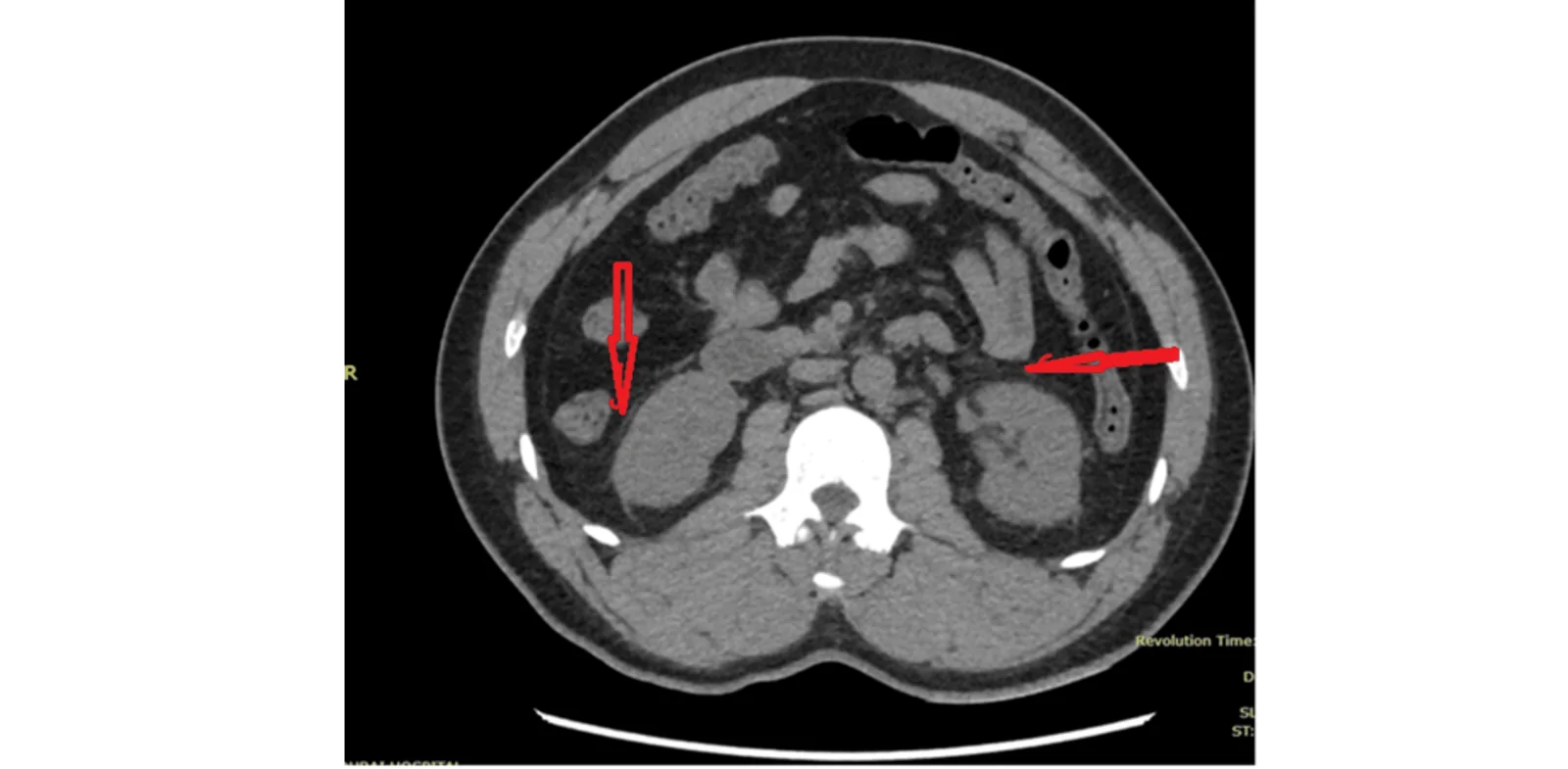
Contrast-enhanced CT of the abdomen revealed perinephric fat stranding and pararenal fascial thickening as pointed by arrows

Contrast-enhanced CT of the abdomen revealed perinephric fat stranding, pararenal fascial thickening, and distended bowel loops without evidence of obstruction or ischemia, as shown in Figure 1.

**Figure 2:**
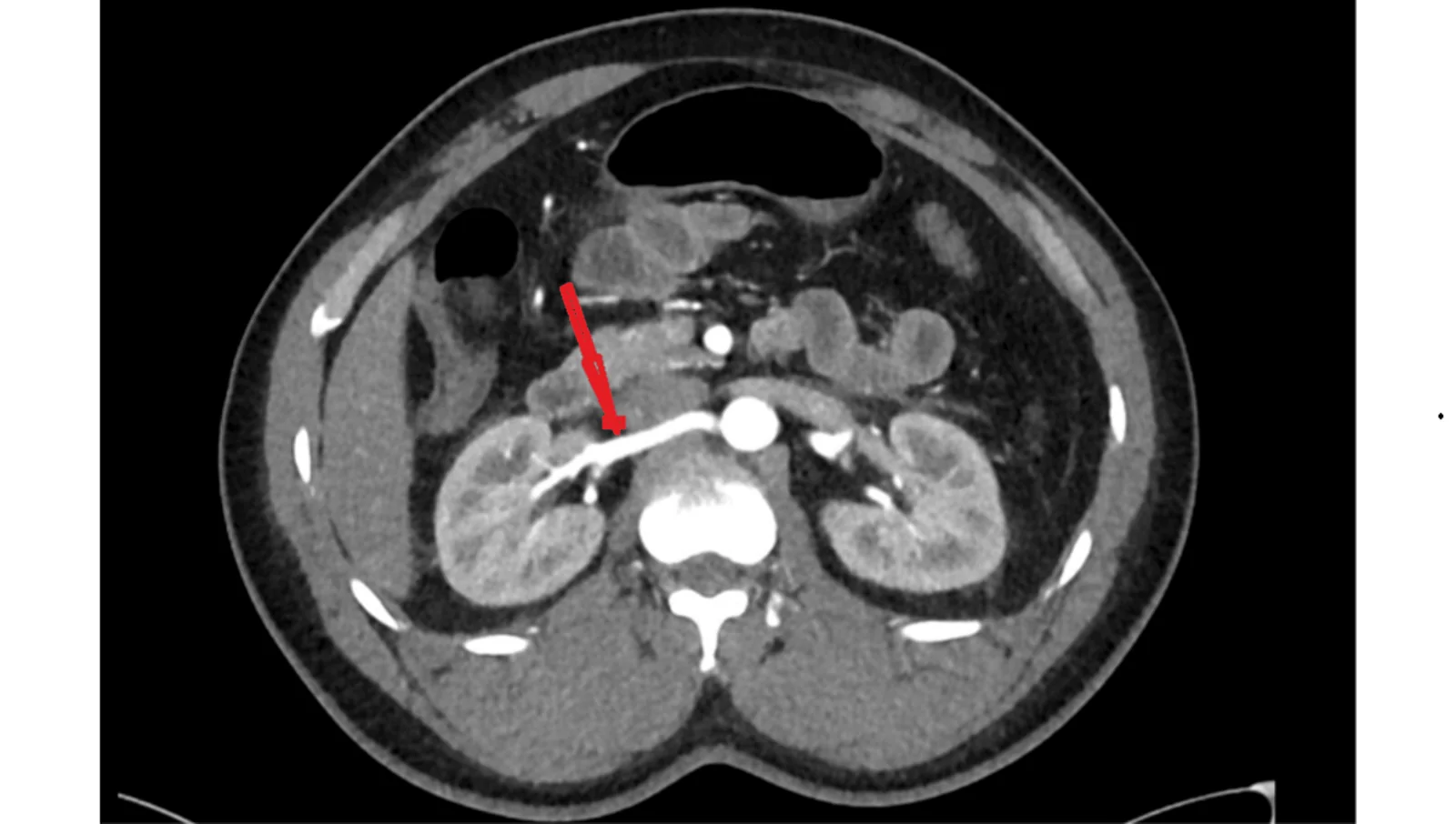
Subsequent CT angiography showing patent renal vasculature (arrows)

Subsequent CT angiography excluded mesenteric ischemia, favoring an infective or inflammatory etiology, as seen in Figure 2.

Treatment

Piperacillin-tazobactam was promptly escalated to intravenous ceftriaxone. The patient required intensive care admission, aggressive fluid resuscitation, vasopressor support, and organ support. He demonstrated rapid clinical and biochemical improvement, with resolution of shock, renal dysfunction, and inflammatory markers. He completed a seven-day course of ceftriaxone and was discharged in stable condition with oral ciprofloxacin for eradication therapy.

Case 2

A previously healthy, 22-year-old man presented in December 2025 to the Emergency Department with acute-onset vomiting following consumption of outside food. He complained of fever, profuse sweating, and generalized myalgia but denied rash, headache, photophobia, abdominal pain, or neurological symptoms. On arrival, he was febrile, hypotensive, and tachycardic. Blood pressure transiently improved with intravenous fluid resuscitation. Initial laboratory investigations revealed severe lactic acidosis, markedly elevated procalcitonin, leukopenia, thrombocytopenia, and acute kidney injury. He was admitted with a provisional diagnosis of septic shock secondary to acute gastroenteritis.

The laboratory investigation for this case is shown in Table 3.

**Table 3:** Laboratory investigation for Case 2 MCV: mean corpuscular volume; MCH: mean corpuscular hemoglobin; MCHC: mean corpuscular hemoglobin concentration; RDW: red blood cell distribution width; APTT: activated partial thromboplastin time; MPV: mean platelet volume; eGFR: estimated glomerular filtration rate; CKD‑EPI: Chronic Kidney Disease Epidemiology Collaboration; SGPT: serum glutamic pyruvic transaminase; ALT: alanine transaminase; ALP: alkaline phosphatase

Test	Reference range	Result
Procalcitonin (PCT)	<0.05 ng/mL	89.00 ng/mL ↑
C‑reactive protein	<5.0 mg/L	42.3 mg/L ↑
WBC count	3.6 – 11.0 10^3/uL	2.5/uL ↓
RBC count	4.50 – 5.50 10^6/uL	4.84/uL
Hemoglobin	13.0 – 17.0 g/dL	14.3 g/dL
Hematocrit	40.0 – 50.0%	42.9%
MCV	77.0 – 95.0 fL	88.5 fL
MCH	27.0 – 32.0 pg	29.6 pg
MCHC	31.5 – 34.5 g/dL	33.4 g/dL
RDW	11.5 – 14.0%	14.7 %↑
Platelet count	150 – 410 10^3/uL	111/uL ↓
Prothrombin time	9.7-11.8 secs	120.o secs ↑
APTT	25.1-37.7	160 ↑
MPV	7.4 – 10.4 fL	8.6 fL
Fibrinogen	200-400 mg/dl	60 mg/dl ↓
Band Neutrophil %	0 – 10%	18 %↑
Nucleated RBCs	0 per 100 WBC	2 per 100 WBC ↑
Neutrophils absolute	2.00 – 7.00 10^3/uL	0.35 /uL ↓
Lymphocytes absolute	1.00 – 3.00 10^3/uL	1.65/uL
Bicarbonate (HCO3)	20 – 28 mmol/L	17.8 mmol/L ↓
Urea	12 – 40 mg/dL	41 mg/dL ↑
Creatinine	0.70 – 1.20 mg/dL	2.06 mg/dL ↑
eGFR (CKD‑EPI)	>60 mL/min/1.73 m²	45.8 mL/min/1.73 m²↓
Lactic acid	0.5 – 2.2 mmol/L	8.83 mmol/L ↑
Troponin T	<14 ng/L	20 ng/L ↑
Bilirubin, total	0 – 1.2 mg/dL	0.74 mg/dL
ALP	40 – 129 U/L	145 U/L ↑
SGPT (ALT)	0 – 41 U/L	32 U/L
Total protein	6.6 – 8.7 g/dL	7.1 g/dL
Albumin	4.4 – 5.1 g/dL	3.9 g/dL ↓
Globulin	2.8 – 3.4 g/dL	3.2 g/dL

The microbiological investigation for this case is shown in Table 4.

**Table 4:** Microbiological investigation for Case 2 BCID PCR: blood culture identification polymerase chain reaction

Microbiological Investigations: Case 2		
Blood culture	Flagged positive within 10 hours	Gram-negative diplococci observed on Gram stain
Blood culture	Positive	Neisseria meningitidis serotype W135 identified
BCID PCR panel	Positive	Neisseria meningitidis detected
Urine Routine	Positive	WBC 5-10, RBC 30-35, hyaline cast 2+
Urine culture	No growth	Not applicable

Treatment: Ceftriaxone IV TID

Shortly thereafter, the patient developed acute agitation followed by rapid hemodynamic collapse and cardiac arrest. Despite immediate advanced cardiopulmonary resuscitation, return of spontaneous circulation was not achieved. Final blood culture identification confirmed *Neisseria meningitidis* serogroup W135, consistent with fulminant meningococcemia.

## Discussion

Meningococcemia presenting predominantly with gastrointestinal manifestations is an uncommon but well-recognized atypical presentation of invasive *Neisseria meningitidis* infection. Patients may initially present with severe abdominal pain, vomiting, diarrhea, or features suggestive of an acute surgical abdomen before the development of the classical manifestations of meningococcal disease, resulting in diagnostic uncertainty and delays in appropriate treatment [1-4]. Several published reports have described patients who were initially evaluated for appendicitis, mesenteric adenitis, or peritonitis before meningococcemia was identified as the underlying cause [3,4].

The pathophysiology of abdominal involvement is not completely understood but is thought to be related to meningococcal endotoxin-induced systemic inflammation, mesenteric vasculitis, intestinal hypoperfusion, and serosal inflammation rather than primary intra-abdominal pathology. In addition to abdominal manifestations, invasive meningococcal disease has been reported with other unusual extra-meningeal presentations, including pericarditis, further emphasizing its diverse clinical spectrum [5]. Recognition of these atypical manifestations is important to avoid unnecessary surgical intervention and, more importantly, to ensure prompt diagnosis and initiation of appropriate antimicrobial therapy.

Both patients in our report lacked the classical features of meningococcal disease, including petechial rash and signs of meningism, highlighting the protean and deceptive nature of meningococcemia. Instead, both presented with features of overwhelming sepsis, including markedly elevated inflammatory markers, thrombocytopenia, lactic acidosis, and rapidly progressive hemodynamic instability. Similar atypical presentations have been reported in the literature, demonstrating that the absence of the characteristic rash or meningeal signs should not exclude meningococcal disease in patients presenting with severe sepsis and unexplained abdominal symptoms [6,7]. Neither patient had a documented history of exposure to a known meningococcal case, recent hospitalization, or previous significant infections.

The markedly different clinical outcomes observed in our patients illustrate the unpredictable course of meningococcemia. Survival in the first patient was likely facilitated by early recognition of sepsis, prompt blood culture collection, rapid molecular identification of N. meningitidis, and timely initiation of targeted antimicrobial therapy together with aggressive supportive care. In contrast, the second patient developed fulminant septic shock with profound leukopenia, severe metabolic acidosis, multiorgan dysfunction, and rapid clinical deterioration despite intensive management. No autopsy was performed, and therefore no autopsy findings or postmortem reports were available for review. Fulminant meningococcemia remains associated with high mortality, particularly in patients presenting with leukopenia, thrombocytopenia, severe metabolic derangements, and circulatory collapse [7].

Serogroup W135 has emerged as an important cause of invasive meningococcal disease and has increasingly been associated with atypical clinical presentations, including predominant gastrointestinal symptoms and septicemia without meningitis [7]. These cases emphasize the importance of maintaining a high index of suspicion for meningococcemia in patients presenting with acute abdominal symptoms accompanied by laboratory evidence of severe sepsis, even in the absence of the classical clinical features.

Early blood culture collection, rapid microbiological diagnosis, and prompt administration of appropriate antimicrobial therapy remain essential to improving outcomes. Increased awareness of these atypical presentations among emergency physicians, surgeons, intensivists, and microbiologists may reduce diagnostic delays and improve survival [8].

## Conclusions

These two cases highlight the diagnostic challenge posed by meningococcemia presenting predominantly with acute gastrointestinal symptoms in the absence of the classical features of meningococcal disease such as rash or meningeal signs. Such atypical presentations may closely mimic acute surgical or intra-abdominal emergencies, potentially delaying recognition and life-saving treatment. Clinicians should maintain a high index of suspicion for invasive meningococcal disease in patients presenting with unexplained severe abdominal pain accompanied by sepsis or septic shock, particularly when no clear intra-abdominal source is identified. Early blood culture collection, rapid microbiological diagnosis, and prompt initiation of appropriate antimicrobial therapy remain critical determinants of survival. Increased awareness of this uncommon presentation may facilitate earlier diagnosis, reduce unnecessary surgical interventions, and ultimately improve patient outcomes.
